# Physical Activity and Sports—Real Health Benefits: A Review with Insight into the Public Health of Sweden

**DOI:** 10.3390/sports7050127

**Published:** 2019-05-23

**Authors:** Christer Malm, Johan Jakobsson, Andreas Isaksson

**Affiliations:** 1 Sports Medicine Unit, Department of Community Medicine and Rehabilitation, Umeå University, 901 87 Umeå, Sweden; christer.malm@umu.se; 2 Department of Molecular Medicine and Surgery, Karolinska Institutet, 171 77 Solna, Sweden; andreas.isaksson@ki.se

**Keywords:** youth, adolescent, elderly, quality of life, relative age effect, exercise, strength and conditioning

## Abstract

Positive effects from sports are achieved primarily through physical activity, but secondary effects bring health benefits such as psychosocial and personal development and less alcohol consumption. Negative effects, such as the risk of failure, injuries, eating disorders, and burnout, are also apparent. Because physical activity is increasingly conducted in an organized manner, sport’s role in society has become increasingly important over the years, not only for the individual but also for public health. In this paper, we intend to describe sport’s physiological and psychosocial health benefits, stemming both from physical activity and from sport participation per se. This narrative review summarizes research and presents health-related data from Swedish authorities. It is discussed that our daily lives are becoming less physically active, while organized exercise and training increases. Average energy intake is increasing, creating an energy surplus, and thus, we are seeing an increasing number of people who are overweight, which is a strong contributor to health problems. Physical activity and exercise have significant positive effects in preventing or alleviating mental illness, including depressive symptoms and anxiety- or stress-related disease. In conclusion, sports can be evolving, if personal capacities, social situation, and biological and psychological maturation are taken into account. Evidence suggests a dose–response relationship such that being active, even to a modest level, is superior to being inactive or sedentary. Recommendations for healthy sports are summarized.

## 1. Introduction

Sport is a double-edged sword regarding effects on health. Positive effects are achieved primarily through physical activity, which is the main part of most sports. Many secondary effects of sport also bring health benefits, such as psychosocial development of both young [1] and old [2], personal development [3], later onset, and less consumption of alcohol [4,5]. Finally, those who play sports have a higher level of physical activity later in life [6], and through sport, knowledge of nutrition, exercise, and health can be developed [7]. Negative effects include the risk of failure leading to poor mental health [8,9], risk of injury [10,11], eating disorders [12], burnout [13], and exercise-induced gastrointestinal tract discomfort [14]. In sport, there are unfortunately also reports of physical and psychological abuse [15]. Negative aspects are more common in elite-level sports, where there is a fine balance between maximum performance and negative health. A somewhat unexpected effect of sport participation is that people submitting to planned training in some cases perform less physical activity compared to those who are exercising without a set schedule. One explanation can be a reduced spontaneous physical activity in the latter group [16]. Because physical activity is increasingly executed in an organized manner [17,18,19], sport’s role in society has become increasingly important over the years, not only for the individual but also for public health.

In this paper, we describe the health effects of sport from a physiological and psychological perspective, related both to physical activity and added values of sport per se. Initially, brief definitions of various concepts related to physical activity and health are given. This is then followed by: (1) A brief description of how physical activity and training affect our body from a physiological perspective; (2) a report on the health effects of physical activity and training; and (3) sport’s specific influences on the various dimensions of health. We chose to discuss the subject from an age-related perspective, separating children/adolescents, adults, and the elderly, as well as separating for sex in each age group.

## 2. Definitions of Physical Activity, Exercise, Training, Sport, and Health

Definitions and terms are based on “Physical activity in the prevention and treatment of disease” (FYSS, www.fyss.se [Swedish] [20]), World Health Organization (WHO) [21] and the US Department of Human Services [22]. The definition of physical activity in FYSS is: “Physical activity is defined purely physiologically, as all body movement that increases energy use beyond resting levels”. Health is defined according to the World Health Organization (WHO) as: “[…] a state of complete physical, mental and social wellbeing and not merely the absence of disease or infirmity” [21].

Physical activity can occur spontaneously (leisure/work/transport) or organized and be divided according to purpose: Physical exercise is aimed primarily at improving health and physical capacity. Physical training is aimed primarily at increasing the individual’s maximum physical capacity and performance [23]. Physical inactivity is described as the absence of body movement, when energy consumption approximates resting levels. People who do not meet recommendations for physical activity are considered physically inactive and are sometimes called “sedentary”. Sport can be organized by age, sex, level of ambition, weight or other groupings [24]. Sport can also be spontaneous [7,17] and defined as a subset of exercises undertaken individually or as a part of a team, where participants have a defined goal [7]. General recommendations for physical activity are found in Table 1, not considering everyday activities. One can meet the daily recommendations for physical activity by brief, high-intensity exercise, and remaining physically inactive for the rest of the day, thereby creating a “polarization” of physical activity: Having a high dose of conscious physical training, despite having a low energy expenditure in normal life due to high volumes of sedentary time. Polarization of physical activity may lead to increased risk of poor health despite meeting the recommendations for physical activity [25,26,27]. During most of our lives, energy expenditure is greater in normal daily life than in sport, physical training, and exercise, with the exceptions of children and the elderly, where planned physical activity is more important [28].

## 3. Aerobic and Muscle-Strengthening Physical Activity

Physical activity is categorized according to FYSS as: (1) Aerobic physical activity and (2) muscle-strengthening physical activity. Physical activity in everyday life and exercise training is mainly an aerobic activity, where a majority of energy production occurs via oxygen-dependent pathways. Aerobic physical activity is the type of activity typically associated with stamina, fitness, and the biggest health benefits [29,30,31]. Muscle-strengthening physical activity is referred to in everyday language as “strength training” or “resistance training” and is a form of physical exercise/training that is primarily intended to maintain or improve various forms of muscle strength and increase or maintain muscle mass [32]. Sometimes, another category is defined: Muscle-enhancing physical activity, important for maintenance or improvement of coordination and balance, especially in the elderly [33]. According to these definitions, muscle-strengthening activities primarily involve the body’s anaerobic (without oxygen) energy systems, proportionally more as intensity increases.

Exercise intensity can be expressed in absolute or relative terms. Absolute intensity means the physical work (for example; Watts [W], kg, or metabolic equivalent [MET]), while relative intensity is measured against the person’s maximum capacity or physiology (for example; percentage of maximum heart rate (%HR), rate of perceived exhaustion (RPE), W·kg^−1^ or relative oxygen uptake in L·min^−1^·kg^−1^ (VO_2_)). In terms of recommendations to the public, as in Table 1, the intensity is often described in subjective terms (“makes you breathe harder” for moderate intensity, and “makes you puff and pant” for vigorous intensity) [27]. While objective criteria such as heart rate and accelerometry will capture the intensity of activity, they may not distinguish between different types of physical activity behaviors [34]. FYSS defines low intensity as 20%–39% of VO_2_max, <40 %HR, 1.5–2.9 METs; moderate intensity as 40%–59% of VO_2_max, 60–74 %HR, 3.0–5.9 METs, and vigorous intensity as 60%–89% of VO_2_max, 75–94 %HR, 6.0–8.9 METs. Absolute intensity, however, can vary greatly between individuals where a patient with heart disease may have a maximal capacity of <3 MET, and an elite athlete >20 MET [35].

## 4. How does the Body Adapt to Physical Activity and Training?

Adaption to physical activity and training is a complex physiological process, but may, in the context of this paper, be simplified by a fundamental basic principle:” The general adaptation syndrome (GAS)” [36,37,38]. This principle assumes that physical activity disturbs the body’s physiological balance, which the body then seeks to restore, all in a dose-related response relationship. The overload principle states that if exercise intensity is too low, overload is not reached to induce desired physiological adaptations, whereas an intensity too high will result in fatigue and possibly overtraining. Thus, for adaptation to occur, greater than normal stress must be induced, interspersed with sufficient recovery periods for restoration of physiological balance [39]. During and immediately after physical exercise/training, functions of affected tissues and systems are impaired, manifested as temporarily decreased performance. You feel tired. In order to gradually improve performance capacity, repeated cycles of adequate overload and recovery are required [40]. In practice, positive effects can be seen after a relatively short period of a few weeks, but more substantial improvements if the training is maintained for a longer period.

As a rule of thumb, it is assumed that all people can adapt to physical activity and exercise, but the degree of adaptation depends on many factors, including age, heredity, the environment, and diet [41,42,43,44]. The hereditary factor (genetics) may be the most critical for adaptation [45]. The degree of adaptation also depends on how the person in question trained previously; a well-trained athlete usually does not have the same relative improvement as an untrained one. Even if training is thought to be specific to mode, intensity, and duration, there are some overlaps. For example, it has been found that strength training in some individuals contributes to a relatively large positive impact on health and endurance, effects previously associated primarily with aerobic exercise [46,47]. The overload principle may, if applied too vigorously in relation to a person’s individual adaptation ability, have detrimental effects, including reduced performance, injury, overtraining, and disease [10]. Training is a commodity that must be renewed; otherwise, you gradually lose achieved performance improvements [48], although some capacities, such as muscle memory, seem to persist for life [49].

General recommendations for health may be stated, but individual predispositions make general training schedules for specific performance effects unpredictable. All exercise training should be adjusted to individual purposes, goals, and circumstances.

## 5. Health Effects of Physical Activity and Training

**Figure 1:**
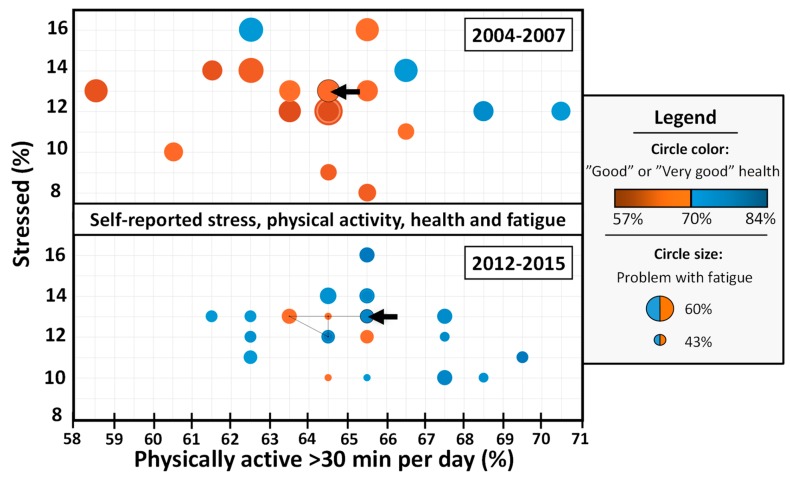
Selected physical and mental health indicators of a Sweden cohort, in relation to the degree of physical activity for the period of years 2004–2007 (*N* = 29,254) and years 2012–2015 (*N* = 38,553). Surveyed subjects are age 16 to 84 years old, with data representing median scores of four years, not normalized for age. Y-axis: Percentage of subjects reporting “stressed”; X-axis: Percentage of subjects indicating physical active at least 30 minutes each day. Each dot represents one County (Län), dot-size indicates self-reported fatigue, and color self-reported healthiness of the County. If 70% of the population states they are having “Good/Very good” health, the dot is blue. If less than 70% states they are having good/very good health, the dot is red. The circle indicated with a black arrow corresponds to nation median. The black line connected to the nation circle represents the movement in the X–Y plane from the year 2004 to 2007, and from 2012 to 2015, respectively. Data retrieved from the Public Health Agency of Sweden 2019-04-22 (www.folkhalsomyndigheten.se).

Human biology requires a certain amount of physical activity to maintain good health and wellbeing. Biological adaption to life with less physical activity would take many generations. People living today have, more or less, the same requirements for physical activity as 40,000 years ago [50,51]. For an average man with a body weight of 70 kg, this corresponds to about 19 km daily walking in addition to everyday physical activity [52]. For most people, daily physical activity decreases, while planned, conscious exercise and training increases [19,53]. Unfortunately, average daily energy intake is increasing more than daily energy output, creating an energy surplus. This is one reason for the increasing number of overweight people, and a strong contributor to many health problems [54]. More sedentary living (not reaching recommended level of physical activity), combined with increased energy intake, impairs both physical and mental capabilities and increases the risk of disease. Despite this, Swedes (as an example) seemed to be as physically active and stressed but had better general health in 2015, compared to 2004 (Figure 1). Compared to 2004–2007, the Swedish population in 2012–2015 reported better overall health (more county-dots are blue) and less fatigue (smaller county-dots) with similar level of physical activity (~65% indicated at least 30 min daily physical activity) and stress (~13% were stressed).

Results in Figure 1 may in part be explained by a polarization of who is physically active: Some individuals are extremely active, others very inactive, giving a similar central tendency (mean/median). As physical activity and mental stress are not changed, but health is, the figure indicates that other factors must be more important to our overall health and fatigue. Recently, a national study of Swedish 11- to 15-year-olds concluded that this age group is inactive for most of their time awake, that is, sitting, standing or moving very little [55]. Time as inactive increased with age, from 67 percent for 11-year-olds to 75 percent for 15-year-olds. The study states that in all age groups, the inactive time is evenly distributed over the week, with school time, leisure time, and weekend. Further, those who feel school-related stress have more inactive time, both overall and during school hours, than those who have less school-related stress.

People active in sports have, in general, better health than those who do not participate in sports, because they are physically and mentally prepared for the challenges of sports, abilities that in many cases can be transferred to other parts of life [56].

However, there is a certain bias in this statement. Sport practitioners are already positively selected, because sickness and injury may prevent participation. As many health benefits of sport are related to the level of physical activity, separation of sport and physical exercise may be problematic. Regardless, societal benefits of these health effects can be seen in lower morbidity, healthier elderly, and lower medical costs [7,57,58].

**Figure 2:**
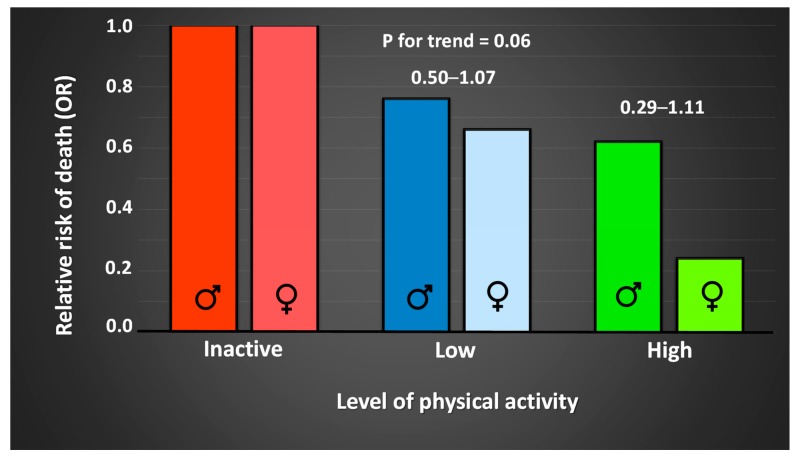
Relative risk (odds ratio; OR) of premature death in relationship to level of physical activity, in 286 male and 148 female twin pairs, adjusted for smoking, occupational group, and use of alcohol [59].

Health effects of physical activity in many cases follow a dose–response relationship; dose of physical activity is in proportion to the effect on health [59,60]. Figure 2 depicts the relationship between risk of death and level of physical activity, in a Finnish twin cohort, adjusted for smoking, occupational group, and alcohol consumption [59]. Odds ratio (OR) for the risk of all-cause mortality in a larger sample in the same study was 0.80 for occasional exercisers (*p* = 0.002, 95% CI = 0.69–0.91). This dose–response relationship between risk of all-cause mortality and physical activity is evident in several extensive studies [60,61,62]. The total dose is determined by the intensity (how strenuous), duration (duration), and frequency (how often). While Figure 2 shows sex differences in death rates, it is likely that sedentary behavior is equally hazardous for men and women, but inconsistent results sometime occur due to inadequate assessment measures, or low statistical power [59,63]. To obtain the best possible development due to physical exercise/training, both for prevention and treatment purposes, a basic understanding of how these variables affect the dose of activity is required, as well as understanding how they can be modified to suit individual requirements. A physically active population is important for the health of both the individual and society, with sport participation being one, increasingly important, motivator for exercise.

There is strong scientific evidence supporting an association between physical exercise/training and good physical and mental health. For example: A reduction in musculoskeletal disorders and reduced disability due to chronic disease [27,64], better mental health with reduced anxiety [65,66], insomnia [67], depression [31], stress [68], and other psychological disorders [69]. Physical and mental health problems are related to an increased risk of developing a number of our major public health diseases and may contribute to premature death (Table 2).

### 5.1. Effects on Physical Health

The effects of physical activity and exercise are both acute (during and immediately after) and long-lasting. Effects remaining after a long period of regular physical activity have far-reaching consequences for health and are described below. For example, some muscle enzymes’ activity can be quickly increased by physical exercise/training but just as quickly be lost when idle [118]. Other changes remain for months or years even if training ends—for instance, increased number and size of muscle fibers and blood vessels [49,119,120]. Good health, therefore, requires physical activity to be performed with both progression and continuity. Most of the conducted physical exercise/training is a combination of both aerobic and muscle strengthening exercise, and it can be difficult to distinguish between their health effects (Table 2).

To describe ill-health, indicators of life expectancy, disease incidence (number), and prevalence (how often) are used [121]. In describing the relationship between physical activity and falling ill with certain diseases, the dose–response relationship, the effect size (the risk reduction that is shown in studies), and the recommended type and dose of physical activity are considered [122]. Table 3 shows the relative effects of regular physical activity ton the risk of various diseases (US Department of Human Services, 2009). The greatest health gains are for people who move from completely sedentary to moderately active lifestyles, with health effects seen before measurable improvements in physical performance. Previously, most scientific studies collected data only on aerobic physical activity. However, resistance exercise also shows promising health (mental and physical) and disease-prevention effects [123,124,125,126,127].

Aerobic physical activity has been shown to benefit weight maintenance after prior weight loss, reduce the risk of metabolic syndrome, normalize blood lipids, and help with cancer/cancer-related side effects (Table 2 and Table 3), while effects on chronic pain are not as clear [29].

Muscle-strengthening physical activity has, in contrast to aerobic exercise, been shown to reduce muscle atrophy [128], risk of falling [75], and osteoporosis [74] in the elderly. Among the elderly, both men and women adapt positively to strength training [129]. Strength training also prevents obesity [130], enhances cognitive performance if done alongside aerobic exercise [131], counteracts the development of neurodegenerative diseases [132,133,134], reduces the risk of metabolic syndrome [135], counteracts cancer/cancer-related side effects [135,136], reduces pain and disability in joint diseases [137], and enhances bone density [137,138]. The risk of falling increases markedly with age and is partly a result of reduced muscle mass, and reduced coordination and balance [76,139,140]. A strong correlation between physical performance, reduced risk of falls, and enhanced quality of life is therefore, not surprisingly, found in older people [141]. Deterioration in muscle strength, but not muscle mass, increases the risk of premature death [142] but can be counteracted by exercise as a dose–response relationship describes the strength improvement in the elderly [122,143]. Recommendations state high-intensity strength training (6–8 repetitions at 80% of 1-repetition maximum) as most effective [144]. Muscle strengthening physical activity for better health is recommended as a complement to aerobic physical activity [29]. Amongst the elderly, vibration training can be an alternative to increase strength [145].

### 5.2. Effects on Mental Health

Mental illness is a global problem affecting millions of people worldwide [147]. Headache, stress, insomnia, fatigue, and anxiety are all measures of mental ill health. The term “*ill health*” constitutes a collection of several mental health problems and symptoms with various levels of seriousness. Studies have compared expected health benefits from regular physical activity for improvement of mental health with other treatments, for example, medication. Most recent studies show that physical activity and exercise used as a primary, or secondary, processing method have significant positive effects in preventing or alleviating depressive symptoms [31,148,149,150,151] and have an antidepressant effect in people with neurological diseases [152]. Training and exercise improve the quality of life and coping with stress and strengthen self-esteem and social skills [69,153]. Training and exercise also lessen anxiety in people who are diagnosed with an anxiety- or stress-related disease [68], improve vocabulary learning [154], memory [155,156], and creative thinking [157].

The same Swedish data as used in Figure 1 show that between the years 2004–2007 and 2012–2015 anxiety, worry, and insomnia decreased but were not obviously correlated to the slightly increased level of physical activity in the population during the same period. Thus, in a multifactorial context, the importance of physical exercise alone cannot be demonstrated in this dataset.

Some of the suggested physiological explanations for improved mental health with physical activity and exercise are greater perfusion and increased brain volume [107,158], increased volume of the hippocampus [106], and the anti-inflammatory effects of physical activity, reducing brain inflammation in neurological diseases [159]. Physical exercise may also mediate resilience to stress-induced depression via skeletal muscle peroxisome proliferator-activated receptor gamma coactivator 1-alpha (PGC-1α), enhancing kynurenine conversion to kynurenine acid, which in turn protects the brain and reduces the risk for stress-induced depression [153]. Further, increased release of growth factors, endorphins, and signaling molecules are other exercise-induced enhancers of mental health [69].

## 6. How Sport Affects Health

Sport’s main purposes are to promote physical activity and improve motor skills for health and performance and psychosocial development [56]. Participants also gain a chance to be part of a community, develop new social circles, and create social norms and attitudes. In healthy individuals, and patients with mental illness, sport participation has been shown to provide individuals with a sense of meaning, identity, and belonging [160,161]. Whether the sport movement exists or not, training and competition including physical activity will happen. Sport’s added values, in addition to the health benefits of physical activity, are therefore of interest. Some argue that it is doubtful, or at least not confirmed, that health development can come from sport, while others believe that healthy sport is something other than health, reviewed in depth by Coakley [162]. In a sporting context, health is defined as subjective (e.g., one feels good), biological (e.g., not being sick), functional (e.g., to perform), and social (e.g., to collaborate) [163]. Holt [56] argued that the environment for positive development in young people is distinctly different from an environment for performance, as the latter is based on being measured and assessed. That said, certain skills (goal setting, leadership, etc.) can be transferred from a sporting environment to other areas of life. The best way to transfer these abilities is, at the moment, unclear.

Having the goal to win at all costs can be detrimental to health. This is especially true for children and adolescents, as early engagement in elite sports increases the risk of injury, promotes one-dimensional functional development, leads to overtraining, creates distorted social norms, risks psychosocial disorders, and has the risk of physical and psychological abuse [15,164]. Of great importance, therefore, is sport’s goal of healthy performance development, starting at an early age. For older people, a strong motivating factor to conduct physical activity is sports club membership [165]. One can summarize these findings by stating sport’s utility at the transition between different stages of the life; from youth to adulthood and from adulthood to old age. There, sports can be a resource for good physical and mental health [166].

**Figure 3:**
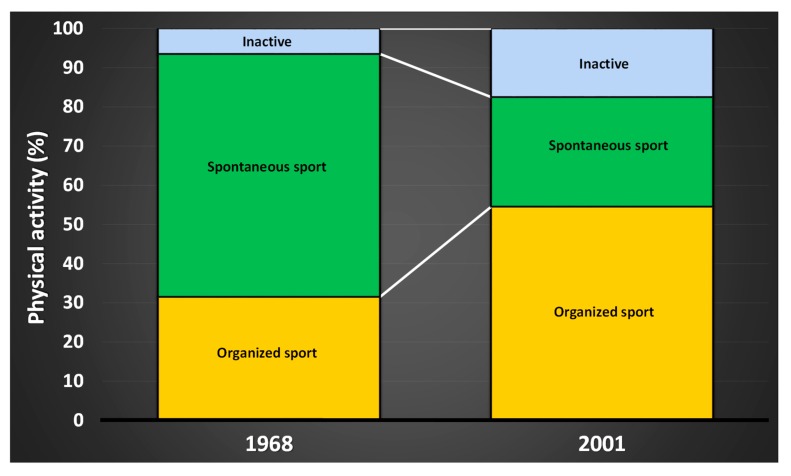
Spontaneous sport has decreased over the last decades, to the advantage of organized sport. Data compiled from Engström, 2004, The Swedish Research Council for Sport Science.

**Figure 4:**
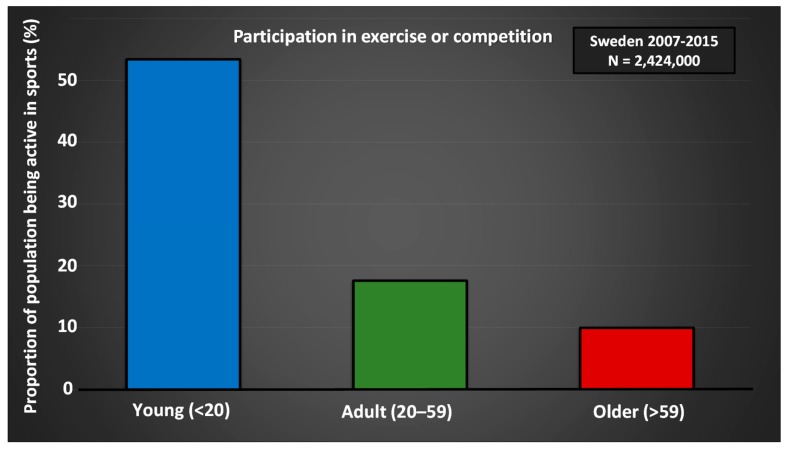
Data compiled from open sources report Sport Statistics (Idrotten i siffror) at The Swedish Sports Confederation for the year 2011 (www.rf.se).

Today, a higher proportion of the population, compared to 50 years ago, is engaged in organized sports, and to a lesser extent performs spontaneous sports (Figure 3), something that Engström showed in 2004 [17] and is confirmed by data from The Swedish Sports Confederation (www.rf.se). Of the surveyed individuals in 2001, 50%–60% of children and young people said they were active in a sports club. The trend has continued showing similar progression to 2011, with up to 70% of school students playing sports in a club. Furthermore, the study shows that those active in sport clubs also spontaneously do more sports [167]. Similar data from the years 2007–2018, compiled from open sources at The Swedish Sports Confederation, confirm the trend with an even higher share of youths participating in organized sports, compared to 1968 and 2001 (Figure 4).

**Figure 5:**
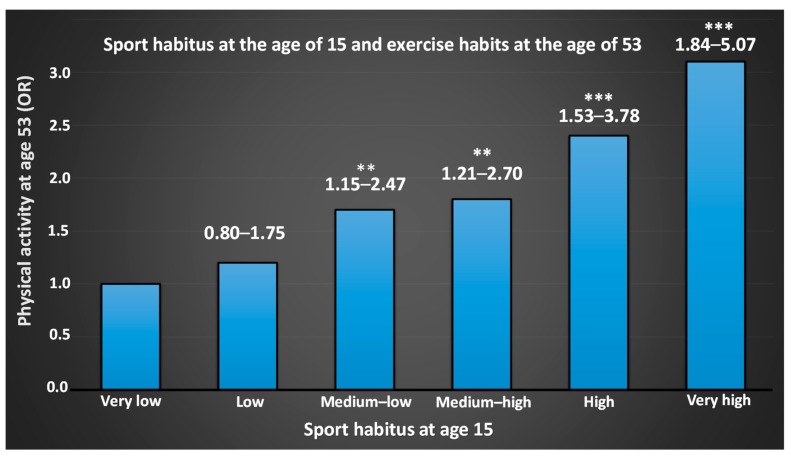
Odds ratio (OR) of physical activity at age 53 in relation to Sport habitus at age 15. Sport habitus (“the total physical capital"), including cultural capital, athletic diversity, and grades in physical education and health are, according to Engström [173], the factors most important for being physically active in later life. For a further discussion on sport habitus, the readers are referred to Engström, 2008 [173]. Numbers above bar show the 95% confidence interval. ** = significant difference from “Very low”, p < 0.01. *** = p < 0.001.

Taking part in sports can be an important motivator for physical activity for older people [165,166]. With aging, both participation in sports (Figure 4) and physical activity in everyday life [168] decreases. At the same time, the number of people who are physically active both in leisure and in organized sports increases (The Public Health Agency of Sweden 2017; www.folkhalsomyndigheten.se). Consequently, among elderly people, a greater proportion of the physical activity occurs within the context of sport [8,28]. Together, research shows that organized sports, in clubs or companies, are more important for people’s overall physical activity than ever before. Groups that are usually less physically active can be motivated through sport—for example, elderly men in sport supporters’ clubs [169], people in rural areas [170], migrants [171], and people with alternative physical and mental functions [172]. No matter how you get your sporting interest, it is important to establish a physical foundation at an early age to live in good health when you get older (Figure 5). As seen in Figure 5, a greater sport habitus at age 15 results in higher physical activity at 53 years of age. Early training and exposure to various forms of sports are therefore of great importance. Participation creates an identity, setting the stage for a high degree of physical activity later in life [173].

## 7. Sport’s Effects on the Health of Children and Young People

The effects of participation in organized sports for children and young people are directly linked to physical activity, with long term secondary effects; an active lifestyle at a young age fosters a more active lifestyle as an adult. As many diseases that are positively affected by physical activity/exercise appear later in life, continued participation in sport as an adult will reduce morbidity and mortality.

It must be emphasized that good physical and mental health of children and young people participating in sport requires knowledge and organization based on everyone’s participation. Early specialization counteracts, in all regards, both health and performance development [174,175].

### 7.1. Positive Aspects

According to several reviews, there is a correlation between high daily physical activity in children and a low risk for obesity, improved development of motor and cognitive skills, as well as a stronger skeleton [176,177]. Positive effects on lipidemia, blood pressure, oxygen consumption, body composition, metabolic syndrome, bone density and depression, increased muscle strength, and reduced damage to the skeleton and muscles are also described [178,179]. If many aspects are merged in a multidimensional analysis [8,173], the factors important for future good health are shown to be training in sports, broad exposure to different sports, high school grades, cultural capital, and that one takes part in sport throughout childhood (Table 4).

Psychological benefits of sports participation of young people were compiled by Eime et al. [1], where the conclusion was that sporting children have better self-esteem, less depression, and better overall psychosocial health. One problem with most of these studies, though, is that they are cross-sectional studies, which means that no cause–effect relationship can be determined. As there is a bias for participating children towards coming from socially secure environments, the results may be somewhat skewed.

### 7.2. Negative Aspects

As Table 4 and Table 5 show, there are both positive and negative aspects of sports. Within children’s and youth sports, early specialization to a specific sport is a common phenomenon [175]. There is no scientific evidence that early specialization would have positive impact, neither for health nor for performance later in life [175]. No model or method including performance at a young age can predict elite performance as an adult. By contrast, specialization and competitiveness can lead to injury, overtraining, increased psychological stress, and reduced training motivation, just to mention a few amongst many negative aspects [174,175]. Another important aspect is that those who are excluded from sports feel mentally worse [8]. As there is a relationship between depressive episodes in adolescence, and depression as adults [116], early exclusion has far-reaching consequences. Therefore, sports for children and young people have future health benefits by reducing the risk of developing depression and depressive symptoms, as well as improved wellbeing throughout life.

**Figure 6:**
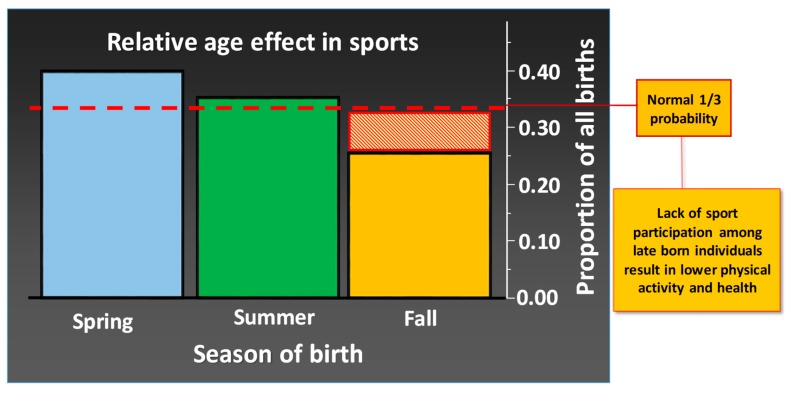
The figure shows the distribution of 7597 children aged 10 years and younger who in 2014 were registered as active in one particular, individual sport in Sweden (data compiled from the Swedish Sport Confederation, www.rf.se). Spring, Summer, and Fall represent January–April, May–August, and September–December, respectively.

While some degree of sport specialization is necessary to develop elite-level athletes, research shows clear adverse health effects of early specialization and talent selection [180]. More children born during the fall and winter (September–December) are excluded [181], and as a group, they are less physically active than spring (January–April) children, both in sports and leisure (Figure 6). In most sports and in most countries, there is a skewed distribution of participants when sorted by birth-date, and there are more spring children than fall children among those who are involved in sport [182,183,184,185,186]. Because a large part of the physical activity takes place in an organized form, this leads to lower levels of physical activity for late-born persons (Malm, Jakobsson, and Julin, unpublished data). Early orientation and training in physical activity and exercise will determine how active you are later in life. Greater attention must be given to stimulating as many children and young people as possible to participate in sport as long as possible, both in school and on their leisure time. According to statistics from the Swedish Sports Confederation in 2016, this relative-age effect persists throughout life, despite more starting than ending with sport each year [18].

When summarize, the positive and negative aspects of sport at a young age can be divided into three categories: (1) Personal identification, (2) social competence, and (3) physiological capacity, briefly summarized in Table 5. A comprehensive analysis of what is now popularly known as “physical literacy” has recently been published [187].

### 7.3. Relevance of Sports

Sports can make children and young people develop both physically and mentally and contribute with health benefits if planned and executed exercise/training considers the person’s own capacities, social situation, and biological as well as psychological maturation. In children and adolescents, it is especially important to prevent sports-related injuries and health problems, as a number of these problems are likely to remain long into adulthood, sometimes for life. Comprehensive training is recommended, which does not necessarily mean that you have to participate in various sports. What is required is diverse training within every sport and club. Research shows that participation in various sports simultaneously during childhood and adolescence is most favorable for healthy and lifelong participation [8,173,188,189].

## 8. Sport’s Effects on the Health of Adults and the Elderly

Adults who stop participating in sports reduce their physical activity and have health risks equal to people who have neither done sports nor been physical [190,191]. Lack of adherence to exercise programs is a significant hindrance in achieving health goals and general physical activity recommendations in adults and the elderly [192]. While several socioeconomic factors are related to exercise adherence, it is imperative that trainers and health care providers are informed about factors that can be modulated, such as intervention intensity (not to high), duration (not too long), and supervision, important for higher adherence, addressed more in depth by Rivera-Torres, Fahey and Rivera [192].

Healthy aging is dependent on many factors, such as the absence of disease, good physical and mental health, and social commitment (especially through team sports or group activities) [193]. Increased morbidity with age may be partly linked to decreased physical activity. Thus, remaining or becoming active later in life is strongly associated with healthy aging [194]. With increased age, there is less involvement in training and competition (Figure 4), and only 20% of adults in Sweden are active, at least to some extent, in sports clubs, and the largest proportion of adults who exercise do it on their own. The following sections describes effects beyond what is already provided for children and youths.

### 8.1. Positive Aspects

Participation in sports, with or without competition, promotes healthy behavior and a better quality of life [166]. Exclusion from sports at a young age appears to have long-term consequences, as the previously described relative age effect (Figure 6) remains even for master athletes (Malm, Jakobsson, and Julin, unpublished data). Because master athletes show better health than their peers [95], actions should be taken to include adults and elderly individuals who earlier in life were excluded from, or never started with sport [195]. As we age, physical activity at a health-enhancing intensity is not enough to maintain all functions. Higher intensity is required, best comprising competition-oriented training [196,197]. One should not assume that high-intensity exercise cannot be initiated by the elderly [198]. Competitive sports, or training like a competitive athlete as an adult, can be one important factor to counter the loss of physical ability with aging [199]. In this context, golf can be one example of a safe form of exercise with high adherence for older adults and the elderly, resulting in increased aerobic performance, metabolic function, and trunk strength [200,201].

### 8.2. Negative Aspects

Increased morbidity (e.g., cardiovascular disease) with aging is seen also among older athletes [202] and is associated with the same risk factors as in the general population [203]. An increased risk of cardiovascular disease among adults (master) compared to other populations has been found [204]. Unfortunately, the designs and interpretations of these studies have been criticized, and the incidence of cardiac arrest in older athletes is unclear [205]. In this context, the difference between competitive sports aiming to optimize performance and recreational sports has to be taken into account, where the former is more likely to induce negative effects due to high training loads and/or impacts during training and games. Although high-intensity training even for older athletes is positive for aerobic performance, it does not prevent the loss of motor units [206].

Quality of life is higher in sporting adults compared to those who do not play sports, but so is the risk of injury. When hit by injury, adults and young alike may suffer from psychological disorders such as depression [207], but with a longer recovery time in older individuals [208]. As with young athletes, secession of training at age 50 years and above reduces blood flow in the brain, including the hippocampus, possibly related to long-term decline in mental capacity [209].

### 8.3. Relevance of Sport

As for children and young people, many positive health aspects come through sport also for adults and the elderly [210]. Sport builds bridges between generations, a potential but not elucidated drive for adults’ motivation for physical activity. The percentage of adults participating in competitive sports has increased in Sweden since 2010, from about 20 percent to 30 percent of all of those who are physically active [18], a trend that most likely provides better health for the group in the 30–40 age group and generations to come.

## 9. Recommendations for Healthy Sport

- 1. Plan exercise, rest, and social life. For health-promoting and healthy-aging physical activity, refer to general guidelines summarized in this paper: Aerobic exercise three times a week, muscle-strengthening exercise 2–3 times a week.
- 2. Set long-term goals.
- 3. Adopt a holistic performance development including physiological, medical, mental, and psychosocial aspects.
- 4. Monitor physiological health over time:
  ○ a. Exercise load (time, intensity, volume);
  ○ b. Recovery (sleep, resting heart rate, appetite, estimated fatigue, etc.);
  ○ c. Sickness (when–where–how, type of infections, how long one is ill, etc.);
  ○ d. Repeat type- and age-specific physical tests with relevant evaluation and feedback;
  ○ e. Frequency of injuries and causes.
- 5. Monitor mental health over time:
  ○ a. Motivation for training, competition, and socializing;
  ○ b. Personal perception of stress, anxiety, depression, alienation, and self-belief;
  ○ c. Repeat type- and age-specific psychological tests with relevant evaluation and feedback.
- 6. Register and interpret signs of overtraining, such as reduced performance over time, while maintaining or increasing exercise load.

## Figures and Tables

**Table 1:** Recommendations regarding physical activity for different target groups. Note that additional health effects can be achieved if, in addition to these recommendations, the amount of physical activity increases, either by increasing the intensity or duration or a combination of both.

Target Group	Recommendations	Purpose
**Children and youth** **Age 6–17 years**	All children and adolescents are recommended at least 60 minutes daily physical activity. Longer is better. The physical activity should be primarily of aerobic nature and the intensity moderate (easy/medium pulse increase) to high (marked pulse increase). Aerobic physical activity at high intensity at least 3 times a week. Muscle-strengthening physical activity 3 times a week. Weight-bearing activity, such as running and jumping, is positive for bone mineral density. The physical activity level will gradually be adapted to the individual’s biological and psychosocial maturation.	Development of muscles and skeletal and nervous system. Maintain a healthy weight and a good mental health. Social development, integration, good self-esteem, and self-confidence. Enhanced learning ability. Recommendations are universal, but for individuals with illness, there may be special recommendations.
**Adults** **Age 18–64**	All adults from 18 years of age and above are recommended to be aerobically physically active at least 150 minutes a week at a moderate intensity (medium pulse increase), or at least 75 minutes per week at vigorous intensity (marked pulse increase). The activities should be distributed over at least three separate days. Muscle-strengthening physical activity at least twice a week should be performed.	Improvements in aerobic work capacity and muscle strength. Recommendations are universal, but for individuals with illness, there may be special recommendations. Profits from carrying out the activity are lower risk of disease, such as disturbed metabolism and certain cancers and bone fractures.
**Elderly** **Age >64**	Same recommendations as adults. Muscle strengthening exercises should be performed at a high velocity, if possible. Balance training should be incorporated prior to aerobic and muscle strengthening training. Individuals with impaired ability should perform as much exercise as possible.	Improvements in aerobic work capacity, muscle strength, and balance. Recommendations are universal, but for individuals with illness, there may be special recommendations. Medical advice may be required before exercise commences. Benefits of carrying out the activity are the same as for adults, and better functional health and independence.

Compiled from FYSS 2017 (www.fyss.se) and WHO 2017 (www.who.int).

**Table 2:** Health-related physiological effects of aerobic and muscle strengthening physical activity. Green circle indicates that the activity contributes with an effect, whereas a red circle indicates that the activity has no proven effect. Orange circle indicates that the activity may in some cases be effective.

Effects on the Body	Health Effects	Aerobic	**Strength**
Larger proportion slow-twitch fibers [70,71]	Lower risk for metabolic syndrome with increased exchange of gases and nutrition [71,72]	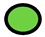	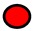
Larger proportion slow-twitch [73]	Increased strength, coordination and balance in elderly [74] and in sickness [75], lower risk for fall [76]	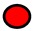	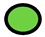
Formation of new capillaries [71]	Increased aerobic capacity [71]	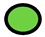	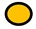
Improved endothelial function [71]	Lower risk for cardiovascular disease [77], improved function in heart disease [78]	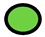	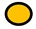
Increased mitochondrial volume [46]	Increased aerobic capacity [79]	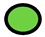	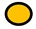
Improved glucose transport [80]	Lower risk or metabolic syndrome/Type-2 diabetes [81]	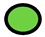	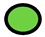
Improved insulin sensitivity [82]	Improved health in people with Type-2 diabetes [82], prevention of Typ-2 diabetes [83]	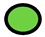	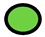
Increased heart capacity [71]	Lower risk for cardiovascular disease [77], fewer depressions [84,85], also in children [86]	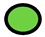	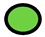
Increased skeletal volume and mineral content [87]	Improved skeletal health [88,89]	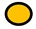	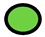
Improved body composition [30]	Lower risk for metabolic syndrome [81]	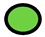	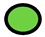
Improved blood pressure regulation [90,91]	Lower risk for cardiopulmonary disease [92]	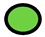	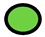
Improved blood lipid profile [93]	Lower risk for cardiopulmonary disease in elderly [94,95] and Alzheimer’s [96] No effect on blood lipid profiles in children and adolescents [97]	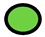	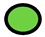
Improved peripheral nerve function [98]	Better coordination, balance and reaction [98,99], especially in children and elderly [100]	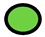	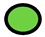
Enhanced release of signaling substances [84,101]	Better sleep [102], less anxiety [68], treatment of depression [31]	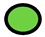	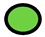
Improved hippocampus function [103]	Improved cognition and memory [104], less medication [103]	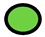	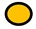
Positive effects on mental capacity [105]	Counteract brain degeneration by diseases [106] and age [107]	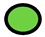	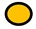
Improved immune function [108]	Decreased overall risk for disease [109,110], anti-inflammatory effects [111,112]	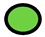	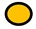
Strengthening the connection between brain, metabolism and immune function [113]	Decreased risk for disease [114], improved metabolism [115], decreased risk for depression [116]	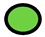	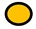
Improved intestinal function [14,113]	Improved health [117], mitigated metabolic syndrome, obesity, liver disease, and some cancers [115]	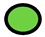	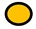

**Table 3:** Disease prevention effects of regular physical activity.

Health Condition	Risk Reduction^1^ or Health Improvement	Recommendations for Physical Activity^2^	Dose-Response Relationship	Differences between Sex, Age, Ethnicity etc.
**All-cause mortality**	30% (44% elderly)	General recommendations	Yes	No
**Cardiovascular disease**	20%–35%	General recommendations	Yes	Insufficient evidence
**Metabolic syndrome**	30%–40%	General recommendations	Yes	No
**Type-2 diabetes**	25%–42%	General recommendations, data primarily on aerobic PA	Yes	Insufficient evidence
**Cancer**	Brain cancer: Limited evidence^2^; Breast cancer: 20%; Bladder cancer: 13%–15%; Colon cancer: 30%; Endometrial cancer: 17%–35%; Esophageal cancer^3^: 6%–21%; Gastric cancer: 19%; Head & neck cancers: 15%–22%, limited evidence; Hematological cancers: No-low effect, limited evidence^3^; Lung cancer: 13%–26%; Ovarian cancer: Limited/conflicting evidence; Pancreatic & prostate cancer: Limited evidence; Renal cancer: 11%–23%; Rectal cancer: No risk reduction, limited evidence; Thyroid cancer: No risk reduction	General recommendations, data primarily on aerobic PA	Renal & thyroid cancer: No. Lung, hematological, head and neck cancers: Limited evidence. Other; Yes.	Breast cancer: Weaker evidence for Hispanic and Black women. Gastric cancer: Weaker evidence for women Renal cancer: Weaker evidence for Asians Lung cancer: Greater effect for women Other: Limited evidence/No known difference
**Overweight and obesity (weight loss)**	PA alone, without diet intervention only has an effect at large volume	General recommendations, combined with diet interventions	Yes	No
**Overweight and obesity (weight maintenance)**	PA supports weight maintenance	General recommendations, stronger evidence for aerobic PA	Limited evidence	Insufficient evidence
**Skeletal health**	36%–68% for hip fracture 1%–2% increased bone density	General recommendations including muscle- strengthening physical activity	Yes	Hip fracture: Largest effect in elderly women Bone density: Largest effect in women
**Muscle mass**	Magnitude is highly variable and mode-dependent	Weight bearing activity	Yes	Decreased effect with age
**Functional strength/capacity (middle age and older)**	30% increased chance to counteract or postpone a decrease in functional strength/capacity 30% lower risk of falls	General recommendations including muscle- and skeletal-strengthening physical activity	Functional health: Yes Falls: No/unclear	Increased functional capacity mostly seen in older adults ages 65 or more.
**Depression**	20%–30% lower	General recommendations	Yes	No
**Sleep**	Improved quality, sleep onset latency and total sleep time	General recommendations	No	No
**Distress**	20%–30% lower	General recommendations	No	No
**Dementia**	20%–30% lower	General recommendations	No	No
**Cognition**	Improved for preadolescent children and adults aged 50 years or older	General recommendations	Conflicting findings	Insufficient evidence for adolescents and adults. Ethnicity: No.

Compiled from US Department of Health and Human Service, https://health.gov/paguidelines/report/ [62,146] ^1^: Risk reduction refers to the relative risk in physically active samples in comparison to a non-active sample, i.e., a risk reduction of 20% means that the physically active sample has a relative risk of 0.8, compared to the non-active sample, which has 1.0. ^2^: In general, general recommendations for PA that are described and referred to herein apply to most conditions. However, in some cases, more specific recommendations exist, more in depth described by the US Department of Health and Human Service, amongst others [62]. ^3^: Evidence is dependent on cancer subtype; refer to US Department of Health and Human Service [62] for in-depth guidance. PA = Physical.

**Table 4:** Compiled health profiles for men and women at the age of 20 years, depending on participation in organized sports at the age of 5, 7, 8, 10, 14, and 17 years.

Physical Activity at Age 20 Years	Girls			Boys		
	Sport Participation as Young					
	Participate	Quit	Never	Participate	Quit	Began late
**Medium/Intense physical activity**	⮉	⮉	⮋	⮉	⮉	⮋
**Current MET**	⇔	⇔	⇔	⮉	⮉	⮋
**Body stature**	⇔	⇔	⇔	⇔	⇔	⇔
**Body weight**	⇔	⇔	⇔	⇔	⇔	⇔
**Fat (%)**	⇔	⇔	⇔	⮋	⮉	⮉
**Fat free mass (kg)**	⮉	⮉	⮋	⮉	⮋	⮉
**Fat free mass index (kg·m^-2^)**	⮉	⮉	⮋	⮉	⮋	⮉
**Physical health (SF-12)**	⮉	⮉	⮋	⇔	⇔	⇔
**Mental health (SF-12)**	⇔	⇔	⇔	⇔	⇔	⇔
**Depression (DASS-21)**	⇔	⇔	⇔	⇔	⮉	⇔
**Anxiety (DASS-21)**	⇔	⇔	⇔	⇔	⇔	⇔
**Stress (DASS-21)**	⇔	⇔	⇔	⇔	⇔	⇔

Classification with repeated latent class analysis creates three groups for girls and boys, respectively: Children who never participated (girls only), participated, quit prematurely, or began late (only boys) in sports. Arrows indicate whether participation in sports at young age has an effect on health at 20 years of age. Green up arrow is positive, red down arrow negative, and a horizontal black double arrow shows that sport had no significant effect. Modified from Howie et. al., 2016 [8].

**Table 5:** Positive and negative aspects with sport (at young age).

Aspect	Positive	Negative
**Personal**	Better self-esteem Better academic results That endurance and hard work pay off Independence and responsibility Making wise decisions Keep a positive attitude Manage stress Set clear goals Higher assessment of skills Higher working standards Better discipline Late alcohol store Lower alcohol consumption (in most sports) Less drugs Greater social capital Better relationships with adults Uses TV/PC less Lower risk of school dropout	Emotional fatigue One-dimensional identity Risk of abuse Increased stress Injuries Temptation for doping Fear of punishment Fear of failure Feeling pressure from the surroundings Fear of disappointing surroundings Risk of burnout Risk of overtraining Poor sleep Decrepit Repeated infections Risk of self-sacrifice Risk of self-injury Increased risk of destructive decisions (doping, cheating etc.) Risk of depression in case of rejection
**Social**	The usefulness of teamwork Good communication Larger contributions to society later in life Larger contributions to the family later in life Lower crime Opportunity in developing countries Increased chance of being active in sports clubs as older Easier to reach with education	Less integrated with the family Social isolation from other society
**Physiological**	Greater physical literacy Abilities to live a healthy life as adult and elderly Less smoking Less drugs Lower body fat Larger muscle mass Beneficial metabolism Higher aerobic and anaerobic capacity Lower risk for fractures as older Reduced general disease risk	Physical fatigue Increased injury risk Risk of eating disorders Overtraining Temptation for doping Risk of abuse (physical and mental) Unilateral training and development For Para athletes, injury can be a double handicap Worse oral health
